# Dark Polymerization Following Partial Radical Photocuring: Effect of Light Intensity and Exposure Duration

**DOI:** 10.1021/acsmaterialsau.5c00225

**Published:** 2026-01-26

**Authors:** Soroosh Farsiani, Frank D. Blum, Hadi Noori

**Affiliations:** a School of Mechanical and Aerospace Engineering, 7618 Oklahoma State University, Stillwater, Oklahoma 74078, United States; b Department of Chemistry, 7618 Oklahoma State University, Stillwater, Oklahoma 74078, United States; c Division of Physical and Computational Sciences, 52456 University of Pittsburgh, Bradford, Pennsylvania 16701, United States

**Keywords:** radical photopolymerization, dark polymerization, UV dosage, light intensity, exposure time, empirical model

## Abstract

Free-radical photopolymerization enables the spatial and temporal customization of the viscoelastic properties of polymers for multifunctional designs. Adjusting light intensity and exposure time can be used to control the kinetics of photopolymerization and, therefore, the nonuniformity in the viscoelastic properties of the polymerized material. Additionally, polymerization that occurs in the dark after partial radical photopolymerization must be taken into account to accurately assess the state of polymerization and maximize photopolymerization efficiency when complete curing is required. In this study, we present a systematic methodology for examining the combined effects of light intensity and exposure time on dark polymerization following partial radical photopolymerization of two acrylate-based photocuring adhesives applicable for bonding dissimilar materials. The findings indicate that light power intensity and material composition are interconnected factors influencing polymerization in the absence of light. Additionally, the results show that, for each material, the stage of photopolymerization—whether in the autoacceleration or autodeceleration phase—significantly affects both the extent and the kinetics of dark polymerization. The similarity of trends in dark radical polymerization, regardless of process and material conditions, led to the proposal of a two-parameter power-law function to empirically describe the trend and rate of dark polymerization.

## Graphical abstract

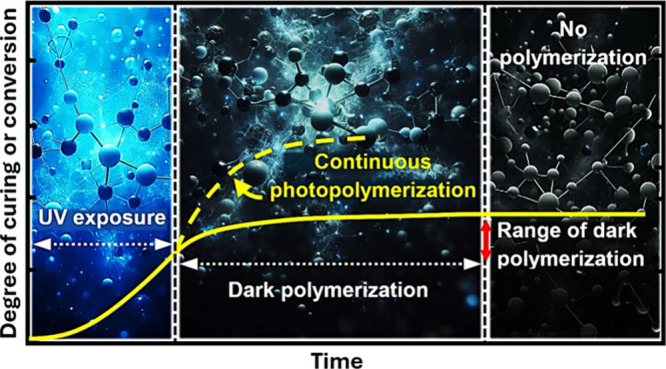

## 1 Introduction

Radical photopolymerization begins with the activation of radical species generated by photoinitiators upon exposure to stimulating light.^1–3^ These radicals react with monomers to initiate polymerization, a time-dependent process that proceeds through chain growth and cross-linking, depending on the monomer functionality. If a sufficient concentration of photoinitiators is present and inhibitors, such as oxygen, are absent, continuous illumination replenishes the polymerizing mixture with new active radicals, while the earlier ones are deactivated by combination or other terminating mechanisms.^1–3^

There are conflicting reports on the occurrence of dark polymerization when the material is shielded from stimulating light after partial radical photocuring. Some studies suggest that polymerization continues,^4–7^ while others indicate that it can cease almost immediately or be statistically insignificant.^8–10^ Considering the differences in experimental procedures and materials reported in the literature,^11–13^ there is a need to understand the effects of curing parameters on the extent and kinetics of dark radical polymerization. These parameters include the chemical composition and macroscopic geometry of the curing material, as well as process parameters such as light intensity and exposure time during partial photopolymerization. Although it has been shown that these parameters have coupled effects on photopolymerization,^3,14^ this study investigates the hypothesis that they will also influence dark radical polymerization.

The use of radical photopolymerization has increased^15^ primarily due to its advantages of being relatively rapid, low-temperature, and low-energy-consuming. In some applications, dark polymerization is favored to enhance mechanical properties,^16,17^ improve uniformity of properties,^8,18^ and reduce light exposure time, thereby decreasing energy consumption.^19,20^ In contrast, preventing dark polymerization is crucial for maintaining the desired heterogeneity required for multifunctional design.^21^ Without dark polymerization, temporal control of polymerization enables the customization of the spatial viscoelastic properties of the polymer,^22,23^ thereby facilitating the imparting of anisotropy in functionality at various length scales. Dark polymerization can be mitigated by quickly terminating living radicals.^23^ Alternatively, the presence of living radicals or the ability to regenerate them without continuous illumination can be key factors in dark polymerization after initial light exposure.^15,24^ For all these applications, a systematic understanding of the extent and rate of dark polymerization after partial photocuring will be necessary to determine the viscoelastic properties of the cured materials.

In this study, we investigated the evolution of dark radical polymerization in two commercially available UV-curable acrylate-based adhesives that differ significantly in their initial viscosities. Using a new experimental procedure to assess dark polymerization, we investigated the effect of UV exposure time relative to the time when the photopolymerization rate was maximum for each material and light intensity. The moment when the photopolymerization rate reached its peak, called t* here, marks the transition between two stages known as autoacceleration and autodeceleration.^14^ Figure 1 shows a typical curve of the degree of curing (DOC) over time during continuous exposure for a radical photopolymerizing material. During light exposure, the curing process may not progress if external or internal inhibiting factors are present. Radical photopolymerizing materials may contain intentionally or unintentionally added inhibitors. For example, dissolved oxygen or exposure of the material surface to oxygen in the surrounding atmosphere inhibits polymerization.^1–3,25^ In commercial materials for radical polymerization, the use of inhibitors, such as 4-methoxyphenol, hydroquinone, and benzoquinone,^25,26^ extends the shelf life of the material. With these internal inhibitors, photopolymerization will be delayed until the inhibitors are consumed. After the inhibition time, called *t*_*inhib*_ in this study, the activated radicals will remain alive long enough to initiate and continue the curing process.

**Figure 1:**
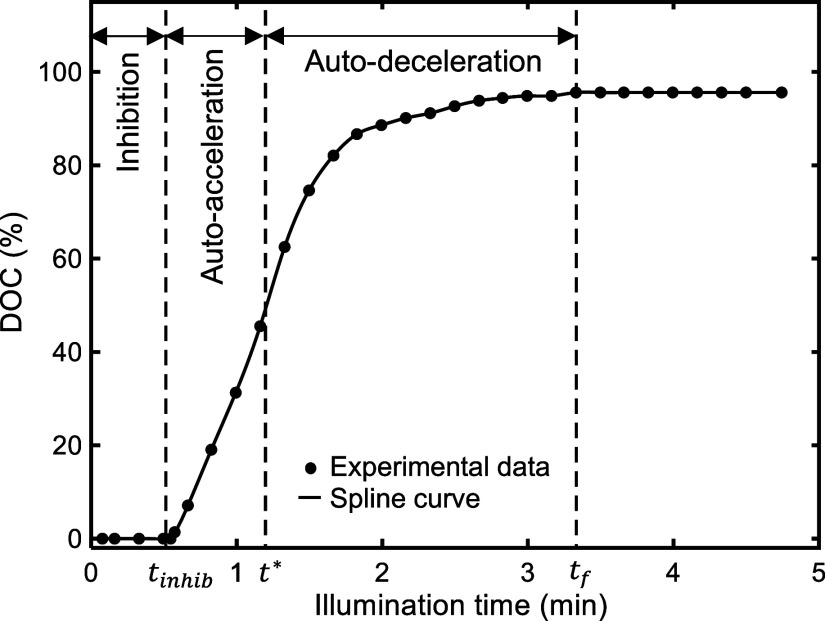
A typical curve for the evolution of bulk radical photopolymerization. The degree of curing or conversion (DOC) data in this figure for material type A used in this study is obtained from the analysis of real-time ATR-FTIR spectroscopy under continuous illumination of 1 mW/cm^2^ UV intensity. The time beyond which the DOC does not change is called *t*_*f*_. The exposure time required before the start of the photopolymerization is called the inhibition time, *t*_*inhib*_.

After the inhibition time, the material cures initially at an increasing rate (autoacceleration mode) before entering the second stage (autodeceleration mode), in which the rate of photopolymerization monotonically decreases until the end of curing at *t*_*f*_. The time denoted by t*, when the photopolymerization rate reaches its maximum, demarks the autoacceleration and autodeceleration modes.

## 2 Methods

### 2.1 Materials

Two commercially available UV-curable adhesives, Type A (Vitralit UV-2885) and Type B (Vitralit UV-2725), were purchased from Panacol (Connecticut, USA) and used to examine the radical dark photopolymerization behavior after different UV exposure conditions. Each of the adhesives was a single-system, solvent-free formulation containing photoinitiators sensitive to UV wavelengths in the 320–390 nm range, with optimal activation at 365 nm. The absorbance spectra for both materials before curing are shown in Figure S1 in the Supporting Information (SI). For these spectra, the UV–vis spectroscopy was conducted using a Cary-5000 (Agilent Technologies, USA) and a 3.5 mL quartz cuvette filled with liquid adhesive. The spectra in the 320–400 nm range were obtained at an average interval of 1.00 nm, with a signal-averaging time of 0.1 s in dual-beam mode and a spectral bandwidth (SBW) of 1.0 nm.

The primary constituents of both adhesives were isobornyl acrylate (25–50 wt %) and acrylic acid (3–5 wt %). Adhesive A also involved 2-hydroxyethyl methacrylate (1–10 wt %), tetrahydrofurfuryl acrylate (3–10 wt %), and maleic acid (1–1.8 wt %). Besides primary constituents, Adhesive B was also composed of 2-ethylhexyl acrylate (1–10 wt %). Other details regarding the composition and concentration of oligomers used to adjust viscosity, and the concentration of the photoinitiator (hydroxycyclohexyl phenyl ketone), are the supplier’s proprietary information.

The steady-shear flow ramp test was conducted using a 40 mm parallel-plate viscometer (Discovery HR 10 rheometer, Waters TA Instruments, USA) with a 0.5 mm gap between the plates at 23 °C. The materials exhibited shear thinning behavior, with initial viscosities of 2150–2010 and 360–300 mPa·s for adhesive types A and B, respectively, across the shear rate range of 1–1000 s^–1^.

### 2.2 Curing and Real-time ATR-FTIR Spectroscopy Procedures

The degree of curing of the adhesives under photo- and dark-polymerization conditions was evaluated using real-time attenuated total reflectance Fourier transform infrared (ATR-FTIR) spectroscopy. All experiments were performed at room temperature (23 °C) using a Nicolet iS50 FTIR spectrometer (Thermo Fisher Scientific, USA) equipped with a diamond crystal ATR accessory.

For each test, a thin layer of adhesive was applied to the diamond crystal, and then a 2 mm-thick fused silica plate was placed on top to minimize oxygen inhibition. A 50 μm-thick piece of Scotch tape with a central hole matching the crystal opening was placed as a spacer between the diamond and the silica plate to ensure a uniform sample thickness of 50 ± 5 μm. The FTIR instrument arm was used to press the silica plate evenly against the adhesive layer before the curing process began. Figure 2 shows the schematic of this configuration. The UV light was irradiated at intensities of 1, 4, 8, and 16 mW/cm^2^, using a 365 ± 5 nm LED source (OmniCure UV LED, Model # AC2110–365, Excelitas Technologies Corp., Ontario, Canada) positioned above the adhesive top surface. The UV light was directed through the silica plate onto the adhesive surface, and its intensity was controlled by adjusting the source-to-sample standoff distance. A minimum distance of 150 mm from the source opening was maintained to ensure passive temperature control. The illumination time was controlled with a resolution of 0.1 s.

**Figure 2:**
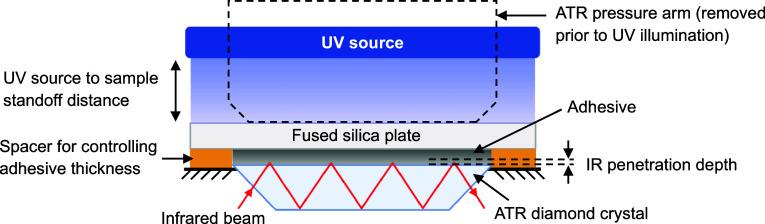
Schematic of the ATR-FTIR device to obtain real-time infrared spectra during curing. The schematic is not on scale.

For all illumination conditions, the UV light irradiance was measured at the adhesive surface using a PD300-TP photodiode sensor (Newport Corporation, Ophir, CA, USA) with a 10 × 10 mm aperture and 0.2 s response time, connected to a StarBright power meter (Newport) for real-time monitoring at a 15 Hz sampling rate. The maximum tolerance of the instantaneous power intensity of the LED source was $\left\{ \begin{array}{c} +0.07\\ -0.00 \end{array}\right.$ mW/cm^2^ for light intensity of 16 mW/cm^2^.

The curing behavior was investigated by comparing the infrared intensities of the carbon–carbon double bonds (C = C) and the carbonyl bonds (C = O) in the system. The former decreases during polymerization, whereas the latter remains constant. Specifically by analyzing the peak height (*H*_*Bond*_) at about 810 cm^–1^ for the C = C twist with respect to the height of the reference peak at about 1720 cm^–1^ for the C = O stretch, the degree of conversion in percentage, *DOC* (%) = [1 – *R*_*t*_/*R*_0_] × 100 was calculated using R = *H*_C=C_/*H*_C=O_ before exposure (*R*_0_) and at the measurement time, *t* (*R*_*t*_). In this paper, the terms ‘degree of curing’ and ‘degree of conversion’ are used interchangeably, and DOC represents both. Any slight shift in the frequency of the C = C and C = O bonds, as well as in their baselines, in ATR-FTIR spectra, was taken into account to accurately measure the degree of conversion. For both adhesive types (A and B), we confirmed that the DOC results from ATR-FTIR experiments were reproducible, with a maximum tolerance of ± 2.7%, by repeating the experiment five times for a single process condition as a representative of all conditions.

For each material and light-intensity level, real-time ATR-FTIR spectroscopy was utilized to monitor both complete and partial photopolymerization, as well as dark polymerization. A chart summarizing the experimental sequence is shown in Figure 3. The analysis of the complete photocuring process provided the t* and *t*_*inhib*_. These results were then used to guide the exposure time during the partial photocuring process, prior to assessing the dark polymerization behavior.

**Figure 3:**
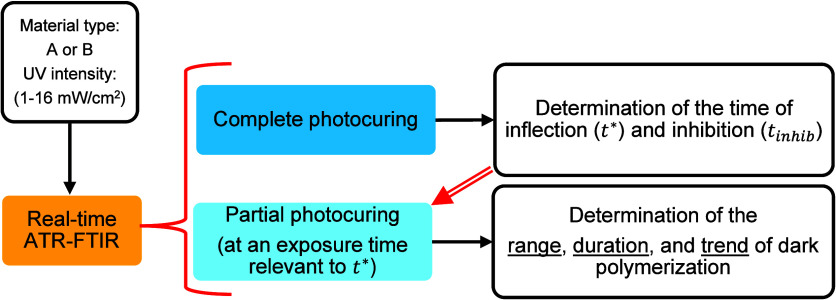
Chart of experiments and analysis of the real-time ATR-FTIR results. The red double-line arrow shows that the t* and *t*_*inhib*_ obtained from complete photocuring were used to guide exposure time for partial photocuring experiments before assessing dark polymerization.

Using the single-sided interferogram setup, spectra were obtained at a resolution of 16 cm^–1^ over the wavenumber range of 600–4000 cm^–1^, with one scan every 0.42 s over a period of 5 min, which was sufficient for complete photopolymerization under all testing conditions. First, by continuously illuminating the sample at each UV light intensity, the time of maximum photopolymerization rate, t*, was determined from the S-shaped curve obtained from the analysis of the ATR-FTIR results. The t* is the time of the inflection point in the S-shaped curve of DOC vs time when the second derivative was zero, and the curvature of the data changed sign.

The t* and the inhibition time (as shown in Figure 1) for each photocuring condition are reported in Figure 4. A power law function (*f*(*P*) = *CP* ^*m*^; *f*(*P*) = *t*_*inhib*_ or t*; – 1 < *m* < 0) very well describes the correlation between these quantities and light power intensity, *P*. The fitting parameters *C* and *m*, as well as the correlation factor, *R* ^2^, are reported in Figure 4.

**Figure 4:**
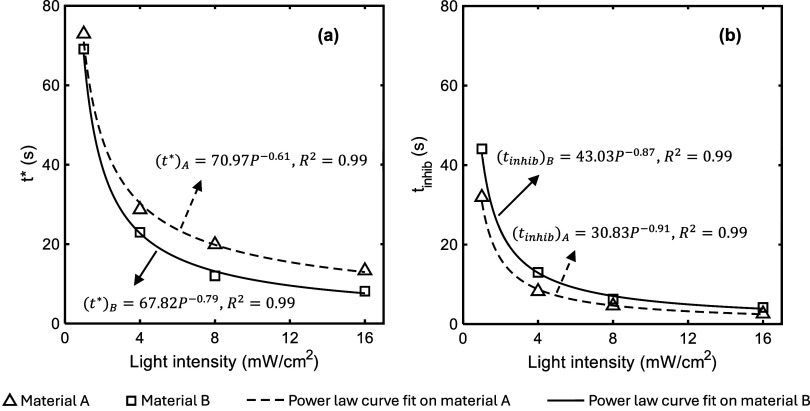
a) The t* and b) *t*_*inhib*_ for the adhesive materials under different levels of UV intensity. The power-law equations fitted to the experimental data, along with the corresponding coefficient of determination, *R* ^2^, are shown on the plot.

A spectral resolution of 16 cm^–1^ was chosen for the real-time ATR-FTIR measurements to enhance temporal resolution and allow faster scan acquisition. This choice was validated by showing that the reduced spectral resolution caused less than a 3% difference in DOC compared to measurements at 4 cm^–1^ spectral resolution for material type B, under UV light intensity of 16 mW/cm^2^ (see Figure 5). This condition yielded the shortest time of inflection and inhibition among all test conditions and was therefore considered the most sensitive to the temporal resolution of ATR-FTIR spectroscopy analysis.

**Figure 5:**
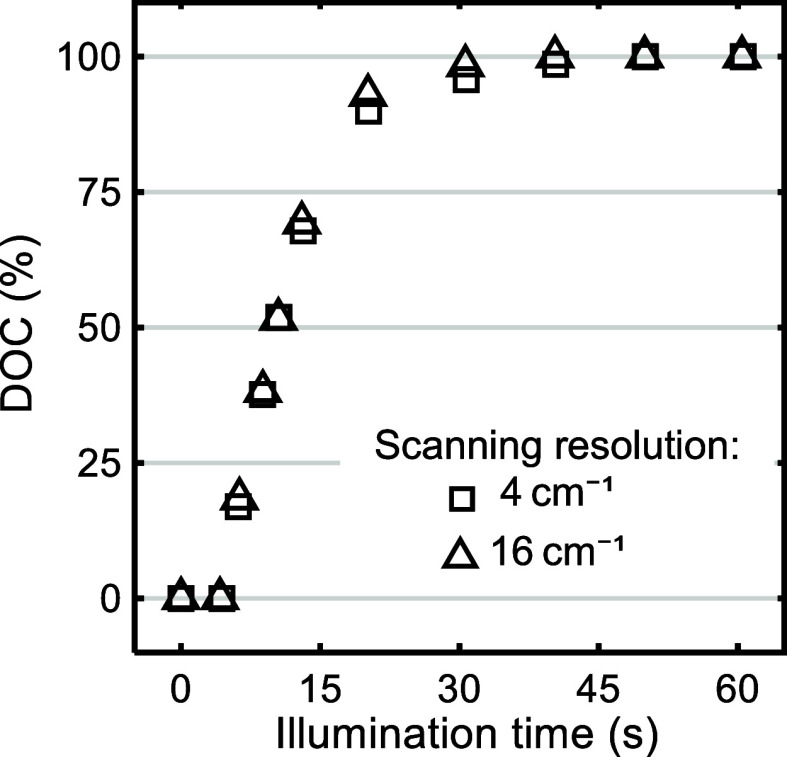
DOC vs illumination time for material type B under 16 mW/cm^2^. These results were obtained from ATR-FTIR spectra recorded at 4 and 16 cm^–1^. This curing condition had the shortest t* and therefore was most susceptible to the lowest accuracy in DOC measurement at scanning resolution of 16 cm^–1^.

To assess the dark polymerization after partial photocuring under each specific UV light intensity, illumination was stopped at a certain illumination time, *t*_*illum*_ = kt* ± 0.1 s, after the start of exposure. The parameter *k* was chosen to halt UV illumination (i) during the autoacceleration regime (*k* = 0.75), (ii) at the time of maximum curing rate (*k* = 1.00), and (iii) during the early and late stages of the autodeceleration regime (*k* = 1.25 and 1.75) of photopolymerization. Real-time ATR-FTIR spectroscopy was performed to monitor the full extent of polymerization during and after photocuring. As an example, Figure 6 shows ATR-FTIR spectra before illumination and at the start (*t*_*illum*_ ≈ t*) and end of dark polymerization (*t*_*f*_) for material type A after partial photopolymerization under UV light intensity of 16 mW/cm^2^.

**Figure 6:**
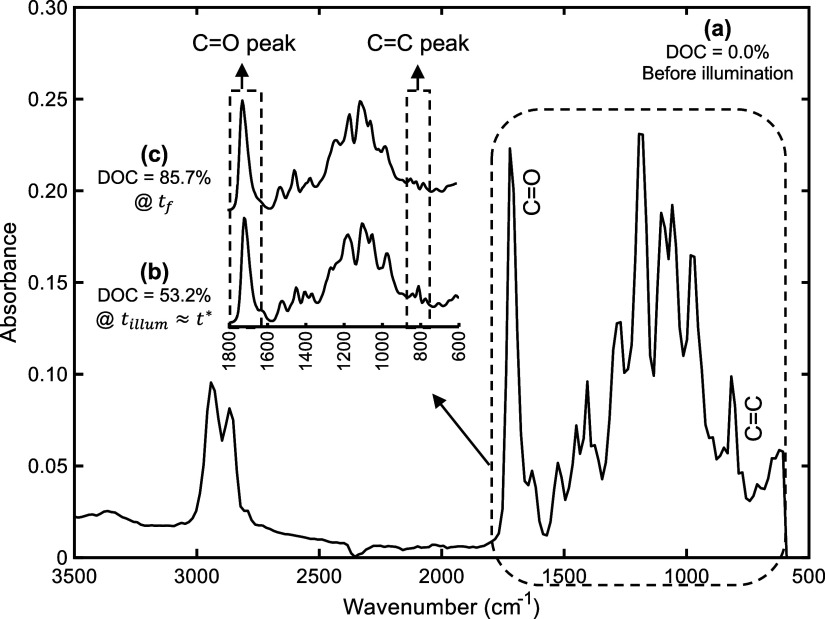
ATR-FTIR spectra acquired at a resolution of 16 cm^–1^ for adhesive type A: a) before illumination, b) at the start of dark polymerization after partial curing (*k* ≈ 1.00) under 16 mW/cm^2^ UV light intensity, and c) at the end of dark polymerization (*t*_*f*_).

## 3 Results and Discussion

Under all testing conditions, regardless of material type, light intensity, or the stage of partial curing before dark polymerization, the trend of the degree of dark polymerization over time was consistent. Following exposure, the rate of dark polymerization steadily decreased until the degree of dark polymerization (*DP*) reached its maximum level at *t*_*f*_ (see Figure 1), beyond which the polymerization effectively ceased. In Figure 7, the curves for the experimental degree of conversion (DOC) are shown. These data were obtained by analyzing real-time ATR-FTIR spectra for the material type A, which was continuously and partially cured under UV light intensity of 1 mW/cm^2^.

**Figure 7:**
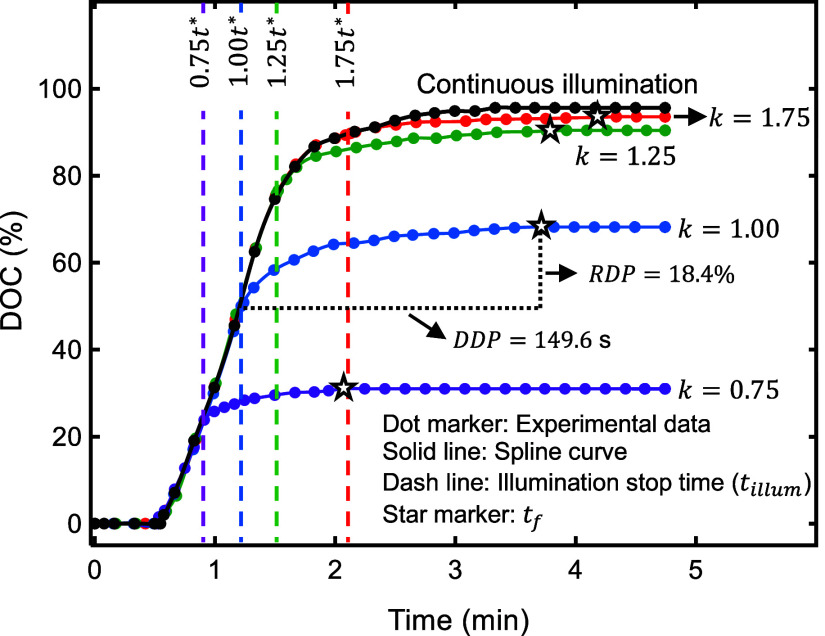
Degree of conversion (DOC) versus time. Dark polymerization is shown after partial photopolymerization before and after the inflection time (t*), which was determined from experiments under continuous illumination. The data in this graph is for material type A under 1 mW/cm^2^. The parameter *k* = *t*_*illum*_/t*. The *t*_*f*_ is shown by a star on each curve. As an example, the range and duration of dark polymerization, RDP and DDP, are shown for *k* = 1.00.

The trend of dark polymerization versus time (*t*), shown in Figure 7, resembles a power-law function of the following form:

$${\left(DP\right)}_{t}=a{\left(\frac{t}{t_{illum}}-1\right)}^{S}+{\left(DOC\right)}_{t_{illum}}$$ 1

where *DP* is the amount of dark polymerization and *a* and *S* are fitting parameters. The power-law function was fitted to the experimental data to find the parameters, *a* and *S*. The coefficient of determination (*R* ^2^) was at least 0.91. This function facilitates the comparison of dark polymerization trends across different test conditions. Notably, the parameter *S* indicates the slope of this function in the log–log scale and parameter *a* = (*DP*)_2*t*_*illum*__ – (*DOC*)*_t_illum__* indicates the amount of dark polymerization when the dark time equals the illumination time (*t*_*i*_).

For all test conditions, the parameters *a* and *S* are presented in Figure 8. Also, the range of dark polymerization, *RDP* = (*DP*)*_t_f__* – (*DOC*)*_t_illum__*, and its duration, *DDP* = *t*_*f*_ – *t*_*illum*_, were analyzed from experimental data. As shown in Figure 9, these parameters were normalized with respect to (*DOC*)*_t_illum__* and *t*_*illum*_ to represent the amount and time of dark polymerization relative to the amount and time of photopolymerization.

**Figure 8:**
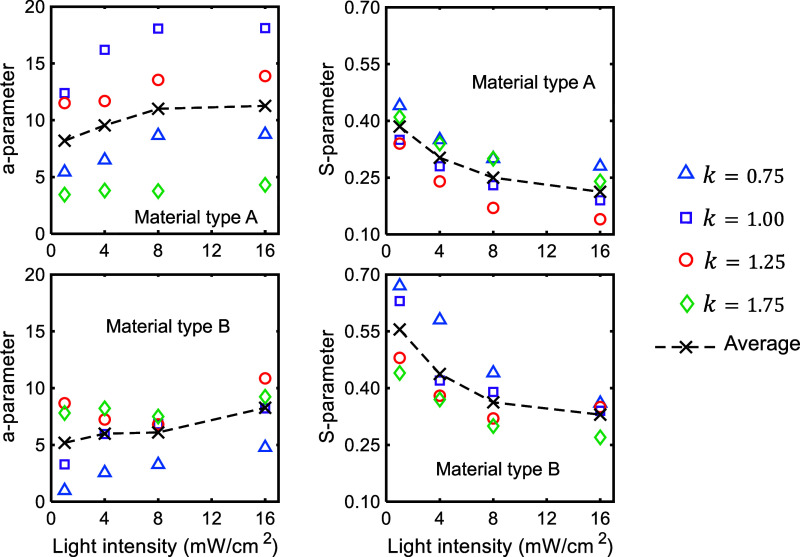
Fitting parameters, a and S, as shown in Equation 1, which describes the trend of the experimental data for dark polymerization after partial photocuring under four different levels of UV light intensity.

**Figure 9:**
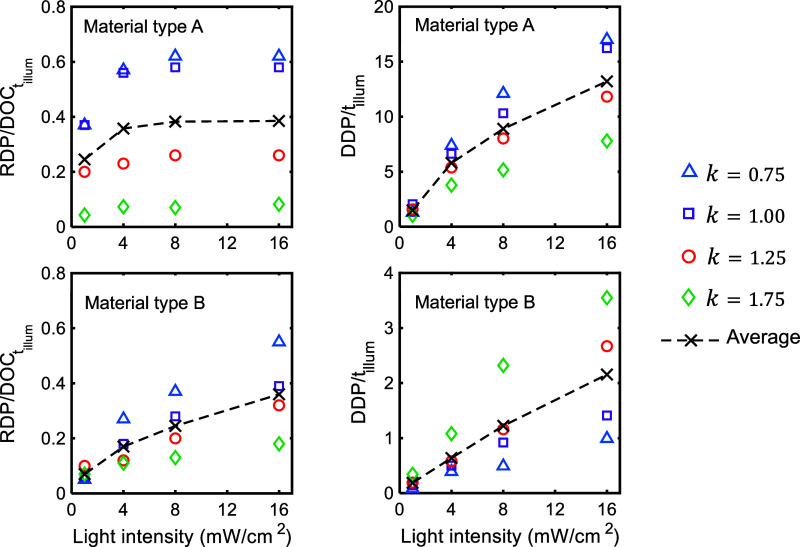
Experimental data for the range (*RDP*) and duration (*DDP*) of dark polymerization normalized by the amount, (*DOC*)*_t_illum__*, and time of photopolymerization (*t*_*illum*_), respectively.

The average of the data for different *k* values at each light intensity in Figures 8 and 9 shows the overall trend of the experimental and modeling parameters with respect to light intensity. For each *k*, Tables 1 and 2 present the average values of the modeling parameters and the experimental data for various UV power intensities. The range of the *a*-parameter is 3.45–18.11 and 0.98–10.86, and the *S*-parameter is 0.44–0.14 and 0.67–0.27 for materials A and B, respectively. The normalized *RDP* is less than 0.62 and 0.55, and the normalized *DDP* is less than 17.00 and 3.55 for materials A and B, respectively.

**Table 1:** Average of Fitting Parameters in Equation (1) and the Range of the Coefficient of Determination for Each *k* Value

		*k* **=** *t*_*illum*_/***t*** *			
Quantity	Material	0.75	1.00	1.25	1.75
**Average of** *a* **-parameter**	A	7.33	16.19	12.66	3.83
	B	2.89	6.07	8.40	8.19
**Average of** *S* **-parameter**	A	0.34	0.26	0.22	0.32
	B	0.51	0.45	0.38	0.35
**Range of** *R* ^**2**^	A	0.96–0.99	0.92–0.97	0.94–0.98	0.95–0.98
	B	0.95–0.99	0.91–0.98	0.93–0.96	0.93–0.97

**Table 2:** Average of Experimental Data for Each *k* Value^a^

		*k* **=** *t*_*illum*_/***t*** *			
Quantity	Material	0.75	1.00	1.25	1.75
**Average of** *RDP*/***DOC*_*illum*_**	A	0.55	0.52	0.24	0.07
	B	0.31	0.23	0.18	0.13
**Average of** *DDP*/***t*_*illum*_**	A	9.44	8.80	6.70	4.44
	B	0.49	0.74	0.94	1.82

a Range of Dark Polymerization (*RDP* ± 0.1%) - Duration of Dark Polymerization (*DDP* ± 0.42 s).

For continuous illumination, the results in Figure 4 show that, regardless of the composition of materials, both inflection time (t*) and inhibition time (*t*_*inhib*_) decrease as light power intensity (*P*) increases. The increase in UV power intensity ensures that inhibitors and photoinitiators farther from the illumination side are reached by stimulating light in a shorter time, thereby expediting the photocuring process in autoacceleration mode. Further study with the objective of assessing the effect of temperature on the photocuring kinetics is required to conclude whether the data trend can be extrapolated to predict the *t*_*inhib*_ and t* for light intensities above 16 mW/cm^2^. The heat dissipation of the UV light source can significantly alter the ambient temperature when the standoff distance between the curing material and the UV light decreases. Such a temperature rise can therefore change the material’s viscosity nonuniformly during a relatively rapid curing process, thereby affecting its photocuring behavior. Future studies on the active cooling process or on the effect of temperature are required to understand the photopolymerization behavior at higher UV power intensities, where environmental temperature may affect the photo- and dark-curing behavior of the polymerizing material.

In this study, the temperature rise due to the curing-induced heating process, in addition to UV source-related heating, was assessed at the highest light intensity of 16 mW/cm^2^, corresponding to the smallest stand-off distance between the source and the adhesive surface. In Figure S2­(a), the schematic of the temperature-monitoring setup shows an adhesive layer, 50 μm thick and 2 × 2 cm^2^ in area, sandwiched between two 40 μm-thick polyethylene terephthalate (PET) films. To minimize the heat transfer through conduction, the sample was held at the periphery of a polymeric hollow cylindrical holder. A K-type thermocouple was taped to the underside of the PET film beneath the adhesive layer. The intensity of UV light was uniform on the sample surface. The results with a 1 s logging interval during the 300 s irradiation time are shown in Figure S2­(b), which illustrates that, during photopolymerization, the temperature first rises from room temperature to a maximum value before gradually decreasing to the temperature level induced by LED source-related heating. The maximum temperature rise during continuous photopolymerization was 14.5 and 10.3 ± 1 °C for materials A and B, respectively. Others reported that for thermally activated polymerization and cross-linking of different acrylate-based monomers, significantly higher temperatures are required.^28–33^ Moreover, the measured temperature rise is unlikely to occur during continuous photopolymerization in the real-time ATR-FTIR experiment, in which the adhesive was in contact with materials such as the metallic casing of the diamond crystal. The metallic casing has a higher thermal conductivity than the PET films used for temperature measurement, and thus a lower maximum temperature is expected during a relatively short photopolymerization time. Therefore, it can be reasonable to neglect the possibility of thermal self-initiation reactions for the radical polymerization of the materials studied in this work. Future research can aim to monitor temperature changes during photo- and following dark-polymerization as a function of material and process parameters to navigate possible changes in the kinetics of photopolymerization and following dark radical polymerization.

For both adhesive systems, comparing the data in Figure 4a,b shows that the difference (time interval) between the *t*_*inhib*_ and t* decreases with light power intensity. Therefore, it is inferred that the polymerization rate in the autoacceleration regime depends on the UV dosage and increases with UV intensity due to the greater number of activated photoinitiators. Consequently, the degree of curing reaches its inflection point sooner under higher power intensity.

Among all conditions, the maximum ratio of *t*_*inhib*_ over t* is 0.64 for material type B under UV intensity of 1 mW/cm^2^, justifying our selection of *k* = 0.75 as a lower limit for partial photocuring in the autoacceleration regime before t*. In this regime, the rate of photopolymerization increases to a maximum level at t*, before declining in the autodeceleration regime, which continues until the material fully cures under continuous illumination. Experimental observations also demonstrated that *k* = 1.75 was a proper choice for partial photocuring before the end of photopolymerization under all test conditions.

In contrast to previous findings for t* of these materials,^14^ material type A consistently showed a longer inflection time, likely due to the difference in curing substrates. In the authors’ previous study,^14^ PET liners were used to sandwich the material for curing. In contrast, this study employed the diamond crystal in the ATR-FTIR apparatus and a plate of fused silica to sandwich the adhesives during photopolymerization and dark polymerization. For future studies, the differences in the optical behavior and adhesion of the substrates can be hypothesized as independent process parameters that significantly influence the kinetics and evolution of radical polymerization.

The *a* and *S* parameters in Equation 1 quantify the trend of dark polymerization, while the range and duration of dark polymerization show its limit and domain. These parameters can therefore be used as a data set to reveal the effects of light intensity and exposure time during the partial photocuring process on the dark polymerization behavior of materials. Despite differences in the chemical composition of the materials in this study, the similarity in their dark polymerization trend is noteworthy. Beyond the scope of this work, future studies could systematically explore the effect of chemical composition on the initial viscosity, photocuring, and following dark polymerization of partially cured materials.

### 3.1 *a*-Parameter and Range of Dark Polymerization

As defined earlier, the *a*-parameter in Equation 1 represents the amount of dark polymerization at a time interval equal to the illumination time. According to the data in Figure 8, this parameter is approximately 1.5–3 times larger for material type A under all testing conditions except for *k* = 1.75. When the illumination time is maximum, this parameter is almost constant for all levels of power intensity, whereas material type B has an average *a*-parameter about twice that of material A. Although *R* ^2^ ≥ 0.91 for all fitting conditions, the modeling work reveals inconsistencies in the results. Notably, the *a*-parameter slightly decreases below 8 mW/cm^2^ at *k* = 1.25 and 1.75.

Regardless of material type, the average of *a*-parameter values shown in Figure 8 increases with light power intensity. However, this increase in the average is not significant for material A at intensity levels above 8 mW/cm^2^, and for material B at intensity levels below 8 mW/cm^2^. The data for the *a*-parameter in Figure 8 suggests that there is likely a *k*-value at which the *a*-parameter values are close to the average. Subject to future work, such a *k*-value may be used as an identifying process parameter to describe the overall dark polymerization behavior of a material with respect to the intensity of the power light that was used for partial photopolymerization.

According to the data in Table 1, the average of values of *a*-parameter for material type A under different light intensities increases with k in the autoacceleration regime (*k* ≤ 1), and decreases in the autodeceleration regime (*k* > 1). For material B, however, the increase in the average of the a-parameter continues to the early stage of the autodeceleration regime. It reaches a maximum of 8.40 at *k* = 1.25 before it slightly decreases to 8.19 at *k* = 1.75, corresponding to the late stage of the autodeceleration regime. The difference between the average a-values is larger for material A with higher initial viscosity. Whether the difference in the *a*-parameter trend with respect to the *k*-parameter can be attributed to the difference in the initial viscosity of these materials remains an open question.

The average of the data in Figures 8 and 9 imply that for a material with spatial nonuniformity in the photocuring stage, i.e., different *k*-values, how the overall dark polymerization changes with light power intensity. Similarly, the data in Tables 1 and 2, including the *a*-parameter and the normalized range of dark polymerization at each *k*-value, can be used to understand the overall dark polymerization behavior if the partial photocuring process was conducted under light with a gradient or nonuniform variation in light intensity. Whether the average analysis method or control of light exposure time can be practically applied to characterize the overall dark polymerization behavior after partial photopolymerization under light with areal nonuniformity in power intensity remains an open question beyond the scope of this work.

The data of the normalized range of dark polymerization, *RDP*/(*DOC*)*_t_illum__*, as reported in Figure 9, indicates that increasing light intensity and decreasing illumination time promote dark polymerization, regardless of the material type. However, material type B, with a lower initial viscosity, exhibits greater sensitivity to these process parameters, except at the lowest light intensity of 1 mW/cm^2^. Under low-intensity exposure, the dark polymerization of material B was insensitive to exposure time, likely due to the fact that the decay of UV intensity through the material’s thickness resulted in a significant decrease in the number of active radicals. Additionally, due to the lower initial viscosity and higher mobility of molecular chains, the radical combination mechanism can more effectively terminate the active radicals and suppress dark polymerization. On the other hand, material A, with a higher level of initial viscosity, did not show appreciable sensitivity to light intensity above 4 mW/cm^2^. It is therefore inferred that controlling dark polymerization for material A (with larger initial viscosity) is more effectively achieved by adjusting exposure time than by varying light intensity. This behavior can likely be attributed to the optical properties of the material under higher levels of light intensity, such that the nonactivated photoinitiators are out of UV reach. Therefore, the number of living radicals at the start of dark polymerization could be comparable for materials photopolymerized under different levels of UV intensity. Additionally, the change in the probability of termination mechanisms for material A under higher levels of UV intensity during photopolymerization is speculated to be another reason, leading to a greater deactivation of the living radicals generated at higher UV intensities during partial curing.

### 3.2 *S*-parameter: Rate of Dark Polymerization

For all testing conditions, regardless of material type and the stage of photocuring, the *S*-parameter shown in Figure 8, an indicator of the overall rate of dark polymerization, decreased with increasing light intensity. The rate of decrease in the *S*-parameter was also reduced with light intensity. This trend in the *S*-parameter is consistent with experimental observations for the normalized amount and duration of dark polymerization, as shown in Figure 9. Regardless of illumination time and material type, the increase in light intensity prolonged dark polymerization, i.e., higher level of light intensity, larger *DDP*/*t*_*illum*_.

The difference between *S*-parameters for different *k*-values at each UV intensity level decreased for material B (converging trend), but increased for material A (diverging trend), with the increase in light intensity (see Figure 8). This observation suggests that the effects of light and illumination time on the rate of dark polymerization are competing and depend on the material type or likely the initial viscosity. For material A, decreasing the light power intensity neutralizes the effect of illumination time on polymerization rate, so the *S*-parameters at different k values become more comparable. Material B, on the other hand, shows an opposite behavior.

The *S*-parameters associated with *k*-values in the range of 0.75–1.25 for material B were larger by 1.3–2.3 times those for material A; however, both materials had comparable *S*-parameter for the longest photopolymerization time (*k* = 1.75) at all four light intensity levels. According to the data for the normalized duration of dark polymerization in Figure 9, material A exhibited approximately one order of magnitude longer dark polymerization with respect to illumination time compared to material B. This observation explains the higher *S* values for material B, which facilitates a combination mechanism for the termination of radicals, for its lower viscosity levels at each stage of curing compared with material A under similar curing conditions.

Increasing the *k*-value decreases the *S*-parameter at each UV intensity level, except for material A at *k* = 1.75, which shows comparable *S*-values to those at *k* = 0.75 (see Figure 8 and Table 2). This exception is considered an artifact. The range of dark polymerization for material A after the longest partial photopolymerization is the lowest among all conditions due to close-to-complete curing at the end of exposure to UV light. Therefore, the rate of dark polymerization will be calculated as high as, or comparable to, that after the shortest exposure time. Ignoring this exception, it is inferred that increasing the illumination time leaves the material more viscous and with higher restriction in the mobility of free radicals. Therefore, a lower polymerization rate is anticipated in the dark.

The normalized dark polymerization time for photopolymerized materials under 1 mW/cm^2^ was insensitive to illumination time, with average amounts of approximately 1.5 and 0.2 for materials A and B, respectively. For UV intensity levels higher than 1 mW/cm^2^, the normalized time of dark curing for material type A decreased with *k*-value. In contrast, that of material B increased with the increase in illumination time, represented by the *k* parameter (see Figure 9 and Table 2).

Comparing the results for these two materials indicates that the effect of material composition or initial viscosity on dark polymerization behavior cannot be neglected. Given the similar compositions of the materials studied, we speculate that the initial viscosity plays a crucial role in the dark polymerization behavior that occurs after partial radical photopolymerization. A lower initial viscosity was associated with a higher rate of dark polymerization, likely due to the increased mobility of active radicals and the relative ease of propagation for polymer chains. However, the termination or annihilation of live radicals via combination mechanisms can account for faster and less dark polymerization. Conversely, materials with higher viscosity showed longer-lasting dark polymerization, though at a slower rate. The ratio of disproportionation to recombination mechanisms for terminating active radicals during dark polymerization is greater in higher-viscosity materials, due to increased restriction in polymer chain mobility.^27^ The probability of the disproportionation mechanism rises due to the caging or trapping of active radicals within an entangled network of chains and cross-links. Further study is therefore required to investigate the effect of viscosity, isolated from composition, on dark polymerization.

### 3.3 Effect of UV Dosage

The dosage of 365 nm UV light is the product of the power intensity received by the photopolymerizing material and the illumination time, *P* × *t*_*illum*_. The duration and range of dark polymerization (*DDP* and *RDP*) with respect to the UV dosage are shown in Figure 10 for both materials. The range of *DDP* and *RDP* is 70.35–215.96 s and 3.83–31.61% for material type A. In comparison, these values are 4.02–50.74 s and 0.42–17.45% for material type B. The data illustrate that the *DDP* and *RDP* levels increase and shift to the right as the light power intensity increases. However, the trends of these characteristics depend on the material type. For material A, both *DDP* and *RDP* are maximum for the dosage associated with *k* = 1.00 when *t*_*illum*_ = t*. For this material, regardless of light power intensity, the *RDP* is at its minimum at the highest UV dosage levels. For power intensities of 1 and 4 mW/cm^2^, the *DDP* is at its minimum at the lowest UV dosage levels. At higher power intensities, the *DDP* trend is more symmetrical, with comparable values at the highest and lowest UV dosages. For material type B, the *DDP* monotonically increases with UV dosage. The *RDP* shows the same trend for conditions at power intensities of 1–8 mW/cm^2^; however, it decreases at the highest intensity level of 16 mW/cm^2^.

**Figure 10:**
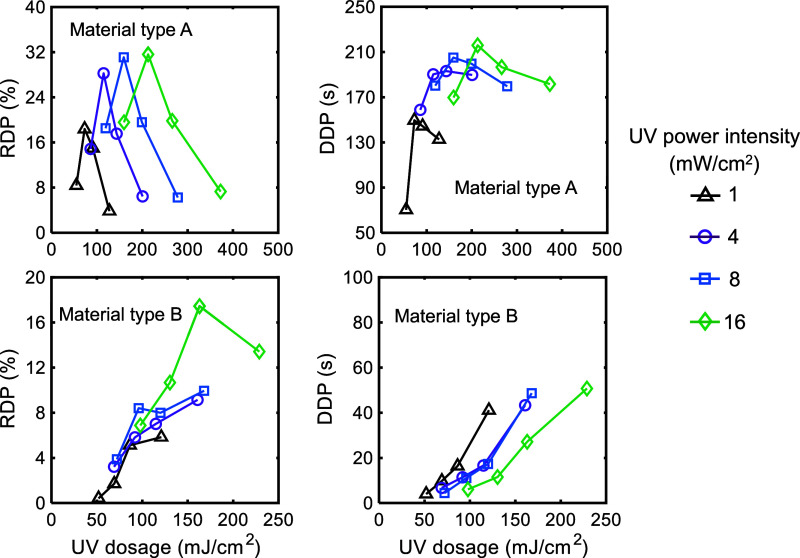
Experimental data for the range (*RDP*) and duration (*DDP*) of dark polymerization with respect to UV dosage.

Considering the similarities in the main composition of these two materials, further research is required to determine whether other material parameters, such as initial viscosity and the type and concentration of components, including the photoinitiator, can explain the difference in their dark polymerization behavior under various process parameters. Nonetheless, the results of this work show how the UV dosage, which is a function of UV power intensity and exposure time, affects radical polymerization in the dark after partial photopolymerization.

## 4 Conclusions

This work presents the results of a systematic study on dark polymerization after partial radical photopolymerization. Guided by the time at which the curing evolution transitions from autoacceleration mode to autodeceleration mode, called t*, the partial photopolymerization was set to have a specified ratio of illumination time (*t*_*illum*_) to t* for every combination of material type and UV light intensity. Therefore, this work presents a systematic methodology for assessing the amount and rate of dark radical polymerization that occurs after partial photocuring. Consequently, the behavior of photopolymerizing materials can be compared more effectively, supporting more efficient process design or the selection of materials for specific applications. In addition, we meticulously analyzed the degree of curing using a real-time ATR-FTIR system to monitor both photopolymerization and dark polymerization. This analysis involved adjusting for slight changes in the C = C and C = O peak positions and their baselines across all spectra to improve the accuracy of degree-of-polymerization measurements.

The similarity of trends in dark radical polymerization, regardless of process and material conditions, enabled the proposal of a two-parameter power-law function to accurately describe the trend and rate of dark polymerization. These parameters were found to have physical significance: 1- The *a*-parameter represents the extent of dark polymerization at a dark time equal to the duration of photopolymerization. 2- The *S*-parameter represents the rate of dark polymerization.

The effects of UV light intensity and exposure time during partial photopolymerization on subsequent dark polymerization are interconnected, and the influence of material type or initial viscosity before curing cannot be ignored. The insights gained from this work can be applied to customizing photopolymerization processes in multistep curing procedures or to saving energy by using dark polymerization as an independent variable in bonding between similar and dissimilar materials.

## Supplementary Material



## Data Availability

The data supporting this study can be obtained upon reasonable request to the corresponding author.

## Notes

Absorbance spectra in the range of 320–400 nm wavelength for material types A and B before curing; schematic of setup for monitoring temperature of a PET (polyethylene terephthalate) substrate during photopolymerization; temperature profiles of the PET substrate in samples with and without adhesive types A and B during continuous photopolymerization under UV intensity of 16 mW/cm^2^ (PDF)

## List of symbols

DOC: Degree of conversion or degree of curing
*a*: A constant coefficient in the power law equation proposed to describe the trend of dark polymerization
*DP*: Degree of dark polymerization
*DDP*: Duration of dark polymerization (s)
*f*(*P*): Power-law function describing the dependence of *t*_*inhib*_ or *t** on UV light power intensity (*P*, in mW/cm^2^)
*H*_*Bond*_: The height of a bond’s absorbance peak obtained from an ATR-FTIR spectrum.
*k*: The ratio of illumination time over time when the rate of photopolymerization reaches its maximum
*RDP*: Range of dark polymerization (%)
*R*_*t*_: The ratio of the heights of the absorbance peak for the C=C bond and the C=O bond obtained from the ATR-FTIR spectrum, used to calculate DOC at a time (*t*)
*S*: Constant power in the power law equation proposed to describe the trend of dark polymerization
*t*_*illum*_: Illumination time
*t*_*inhib*_: Inhibition time
*t*_*f*_: The time at the end of polymerization
*t**: Time of inflection point on the photopolymerization S-curve; the time at which the photopolymerization rate reaches its maximum
