# Supplementary material for: Dark Polymerization Following Partial Radical Photocuring: Effect of Light Intensity and Exposure Duration
Source: ACS Mater Au. 2026 Jan 26;6(3):566–75. doi: 10.1021/acsmaterialsau.5c00225 (PMC13177412; doi:10.1021/acsmaterialsau.5c00225)
Supplement: Supplementary file 1 [file mg5c00225_si_001.pdf]

Supporting Information

Dark Polymerization Following Partial Radical Photocuring: Effect of Light Intensity and Exposure Duration

Soroosh Farsiani [a], Frank D. Blum [b], Hadi Noori [c*]
[a] *School of Mechanical and Aerospace Engineering, Oklahoma State University, Stillwater, OK 74078, United States*
[b] *Department of Chemistry, Oklahoma State University, Stillwater, OK 74078, United States*
[c] *Division of Physical and Computational Sciences, University of Pittsburgh, Bradford, PA 16701, United States*
[*] Email: hadi.noori@pitt.edu

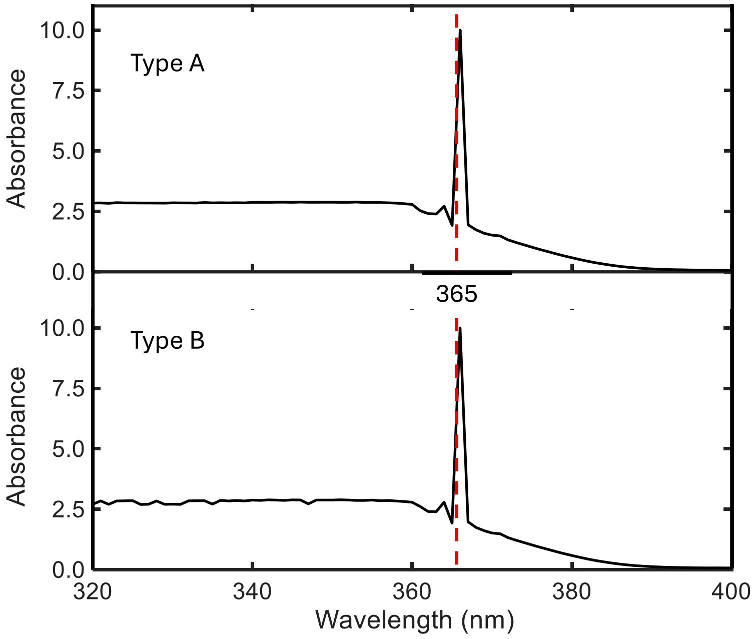

Fig. S1. Absorbance spectra in the range of 320-400 nm wavelength for material types A and B before curing.

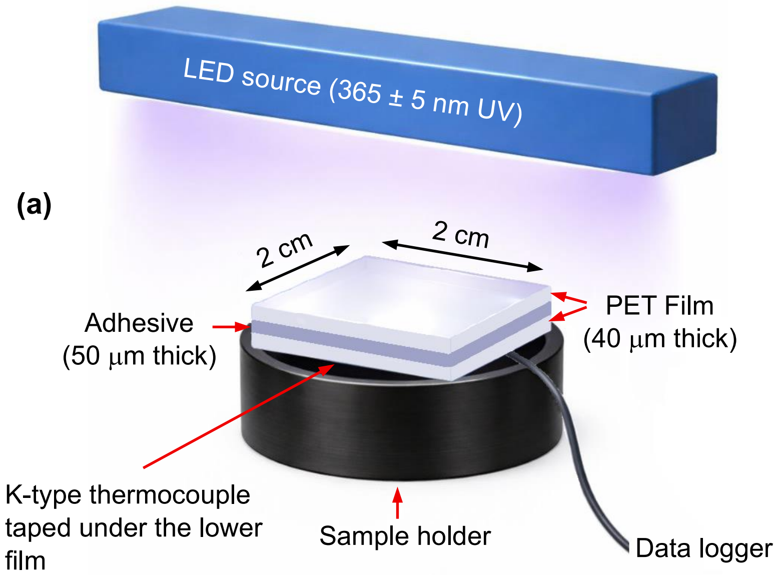

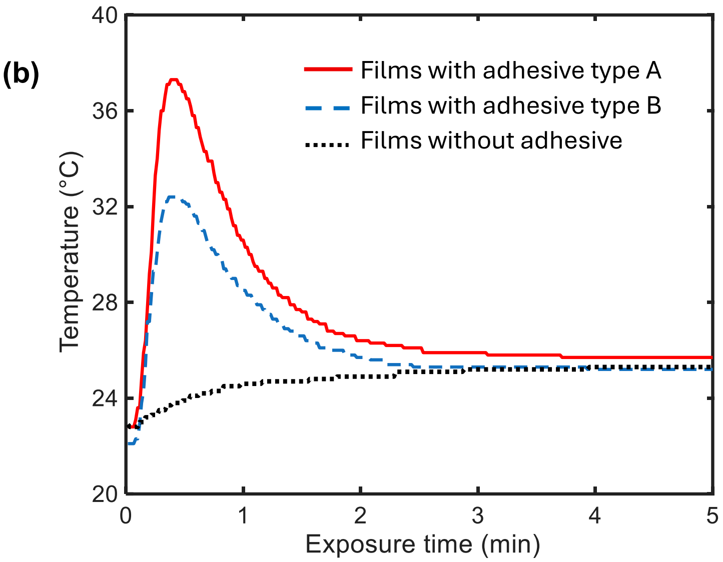

Fig. S2. (a) Schematic of setup for monitoring temperature of a PET (polyethylene terephthalate) substrate during photopolymerization, (b) temperature profiles of the PET substrate in samples with and without adhesive types A and B during continuous complete photopolymerization under UV intensity of 16 mW/cm^2.
